# Supplementary material for: Desiccation resistance differences in Drosophila species can be largely explained by variations in cuticular hydrocarbons
Source: eLife. 2022 Dec 6;11:e80859. doi: 10.7554/eLife.80859 (PMC9757832; doi:10.7554/eLife.80859)
Supplement: Supplementary file 1. — (a) Phylogenetic signals for mbCHCs in Drosophila species. (b) Phylogenetic signals for desiccation resistance in Drosophila species. (c) Summary of the Phylogenetic Generalized Linear Square (PGLS) models between the longest mbCHCs and desiccation resistance for females and males in 50 Drosophila and related species. (d) List of species used in this study. [file elife-80859-supp1.docx]

**Supplementary File 1a.** Phylogenetic signals for mbCHCs in *Drosophila* species.

| **Sex** | **2MeC24** | **2MeC26** | **2MeC28** | **2MeC30** | **2MeC32** | **Pagel’s *λ*** |
| --- | --- | --- | --- | --- | --- | --- |
| Female | 92.2% | 87.9% | 90.9% | 90.4% | 88.8% | 0.75 |
| Male | 93.1% | 90.1% | 91.9% | 91.8% | 92.0% | 0.82 |

**Supplementary File 1b.** Phylogenetic signals for desiccation resistance in *Drosophila* species.

| **Sex** | **Pagel’s *λ*** | **LogLikelihood (*λ)*** | **Likelihood_Ratio (*λ* = 0)** | ***P*-value** |
| --- | --- | --- | --- | --- |
| Female | 0.91 | -22.4 | 45.2 | < 0.001 |
| Male | 0.97 | -25.4 | 61.7 | < 0.001 |

**Supplementary File 1c.** Summary of the Phylogenetic Generalized Linear Square (PGLS) models between the longest mbCHCs and desiccation resistance for females and males in 50 *Drosophila* and related species.

| **Sex** | **Term** | **t value** | **P value** |
| --- | --- | --- | --- |
| Female | Length | 3.2 | 0.002 |
|  | Quantity | -3.6 | < 0.001 |
|  | Interaction | 3.5 | < 0.001 |
| Male | Length | 1.9 | 0.08 |
|  | Quantity | -2.3 | 0.03 |
|  | Interaction | 2.3 | 0.03 |

**Supplementary File 1d.** List of species used in this study

| **Genus** | **Species** | **Sources and strain code from NDSSC** |
| --- | --- | --- |
| *Drosophila* | *D. mojavensis* | 15081-1352.10 |
| *Drosophila* | *D. arizonae* | 15081-1271.41 |
| *Drosophila* | *D. aldrichi* | 15081-1251.23 |
| *Drosophila* | *D. mulleri* | 15081-1371.01 |
| *Drosophila* | *D. buzzatii* | 15081-1291.63 |
| *Drosophila* | *D. mercatorum* | 15082-1521.38 |
| *Drosophila* | *D. repleta* | 15084-1611.13 |
| *Drosophila* | *D. americana* | 15010-0951.00 |
| *Drosophila* | *D. novamexicana* | 15010-1031.14 |
| *Drosophila* | *D. lummei* | 15010-1011.01 |
| *Drosophila* | *D. virilis* | 15010-1051.87 |
| *Drosophila* | *D. littoralis* | 15010-1001.11 |
| *Drosophila* | *D. lacicola* | 15010-0991.13 |
| *Drosophila* | *D. borealis* | 15010-0961.00 |
| *Drosophila* | *D. montana* | 15010-1021.23 |
| *Drosophila* | *D. flavomontana* | 15010-0981.00 |
| *Drosophila* | *D. nasuta* | 15112-1781.00 |
| *Drosophila* | *D. albomicans* | 15112-1751.00 |
| *Drosophila* | *D. sulfrigaster* | 15112-1811.04 |
| *Drosophila* | *D. immigrans* | 15111-1731.03 |
| *Drosophila* | *D. equinoxialis* | 14030-0741.00 |
| *Drosophila* | *D. paulistorum* | 14030-0771.11 |
| *Drosophila* | *D. willistoni* | 14030-0811.24 |
| *Drosophila* | *D. nebulosa* | 14030-0761.06 |
| *Drosophila* | *D. prosaltans* | 14045-0901.07 |
| *Drosophila* | *D. saltans* | 14045-0911.01 |
| *Drosophila* | *D. sturtevanti* | 14043-0871.16 |
| *Drosophila* | *D. azteca* | 14012-0171.03 |
| *Drosophila* | *D. affinis* | 14012-0141.02 |
| *Drosophila* | *D. persimilis* | 14011-0111.46 |
| *Drosophila* | *D. pseudoobscura* | 14011-0121.94 |
| *Drosophila* | *D. bipectinata* | 14024-0381.21 |
| *Drosophila* | *D. ananassae* | 14024- 0371.13 |
| *Drosophila* | *D. serrata* | 14028-0681.00 |
| *Drosophila* | *D. kikkawai* | 14028-0561.14 |
| *Drosophila* | *D. birchii* | 14028-0521.00 |
| *Drosophila* | *D. elegans* | Gift from the P. Wittkopp Lab (U. Michigan) |
| *Drosophila* | *D. gunungcola* | Gift from the P. Wittkopp Lab (U. Michigan) |
| *Drosophila* | *D. biarmipes* | 14023-0361.09 |
| *Drosophila* | *D. suzukii* | Gift from the R. Isaacs Lab (MSU) |
| *Drosophila* | *D. erecta* | 14021-0224.01 |
| *Drosophila* | *D. teissieri* | 14021-0257.01 |
| *Drosophila* | *D. yakuba* | 14021-0261-01 |
| *Drosophila* | *D. mauritiana* | 14021-0241.151 |
| *Drosophila* | *D. simulans* | W501 (14021-0251.195) |
| *Drosophila* | *D. melanogaster* | Gift from the S. Carroll Lab (U. Maryland) |
| *Scaptodrosophila* | *S. latifasciaeformis* | 11030-0061.01 |
| *Scaptodrosophila* | *S. lebanonensis* | 11010-0011.00 |
| *Scaptodrosophila* | *S. rufifrons* | 11040-0071.00 |
| *Chymomyza* | *C. procnemis* | 20000-2631.01 |
